# New York Odontological Society

**Published:** 1890-09

**Authors:** 

**Affiliations:** New York Odontological Society


### NEW YORK ODONTOLOGICAL SOCIETY.

(Continued from page 472.)

*Dr. S. E. Davenport.*—If I may be allowed, I would like to read an extract from a personal letter recently received from Dr. George H. McCausey, of Janesville, Wis., who, it will be remembered, was the author of a very valuable paper which was read before this society last January, the title of which was "The Dental Pulp and its Relation to the other Tooth Tissues." Dr. McCausey says,—

"On reading the discussion upon my paper I was impressed with an idea, more than ever, of the advantage to be derived by both hearers and the author when a paper is read by the writer himself. Many questions arise which the writer cannot or does not always foresee, and which, if present, he may perhaps be able to render clear.

"The microscopist always encounters a certain amount of difficulty in the demonstration of histological truths before a miscellaneous body of men who are not microscopists, even if he has his microscope and slides present. My own observation and experience have taught me the folly of submitting the finest differentiated specimens to untrained eyes even with low powers, to say nothing of those sufficiently high for use in a study of the tissues of the pulp.

"I have always found myself better able to instruct my hearers through the medium of photo-micrographs than through the microscope itself, except in the case of the experienced observer. No one should expect to accomplish much in microscopy unless he is willing to take the time and trouble to render himself familiar with the technology of cutting and mounting sections. He must study the necessities in each variety of tissue, or he will be heard complaining that his friends arrive at results which are very much different from those which he arrives at. One cannot buy the slides which are generally placed upon the market and study them to any great advantage. Their secrets are not yielded up readily, for they are behind a balsam door which has been locked with the key alcohol. Since sending the paper to your society I have done little with the microscope on account of my health and have written none for the same reason.

"The tissues which I studied while writing the paper I did not mount for lack of time, but when I make some more sections of the tissues I will try to get the time to prepare a slide for Dr. Allan, and which I hope will please him on account of what the microscope may reveal in it. In the mean time, I think that it would pay him to visit Dr. Heitzmann in his laboratory and look his slides carefully over, and he will be able to see the reticulum of 'living matter' even as Dr. Heitzmann himself puts it.

"At one time I doubted its existence, but afterwards found it to be on account of my faulty methods both in staining and mounting. Objectives of the very best defining power are necessary, or indifferent results will be obtained. I threw away about all my leisure time during a period of about six months in an endeavor to combine the use of objectives of not the best correction, with faulty methods in preparation of tissue. I am greatly pleased at the kindly reception of my paper by the Society."

*Dr. Allan.*—Mr. President, Drs. Andrews and Sudduth have both examined the preparations in question and both have failed to see anything approaching in appearance the reticulum described. I have examined very carefully, and I want to say that there is no reticulum to be seen in them; of that I am certain; and I state here now, publicly and in dead earnest, that if any man will get me slides, authenticated by Professors Heitzmann and Abbott, saying that they contain the reticulum, and also the privilege of publishing precisely what I see, with illustrations, and the criticisms of experts, I will take it as a very great favor.

*The President.*—We will now pass to the reading of the paper of the evening, and I have the pleasure of introducing Dr. L. Duncan Bulkley.

(For Dr. Bulkley's paper, see pages 449 and 513.)

*The President.*—The subject of Dr. Bulkley's very valuable and interesting paper is before you and is open for discussion. We are favored by having present several eminent medical men this evening, and the society would be very glad to hear from them, and also from any dentists who are our guests, either in discussing the subject or asking questions. It occurs to me that Dr. Bulkley has opened the way for the dentists present to draw out considerably more information. Dr. Morrow is present; we would be glad to hear from him.

*Dr. P. A. Morrow.*—Mr. President and gentlemen, the subject has been so very fully and exhaustively presented by the reader of the paper as to leave little to be added in the way of discussion.

Considering the multitudinous chances of contagion to the dentist in his work, it is somewhat remarkable that so few cases of syphilis communicated to the operator have been recorded. This may be due to the fact, however, that dentists as well as physicians contracting contagion in this way are not very ambitious to advertise themselves as subjects of syphilis. I have no doubt that a great many cases of syphilis communicated in the exercise of professional duties have not been reported, for the reason that by the general public, as well as by a great portion of the profession, syphilis is looked upon as a shameful disease,—a disease which carries with it the stamp of immorality. From the great number of cases of non-venereal syphilis, which Dr. Bulkley has referred to, it is very evident that syphilis is not necessarily a venereal disease. Fournier, in the latest edition of his work on "Syphilis and Marriage," states that he has investigated the histories of several hundred cases of syphilitic women, and he found that one out of every five of these patients was a married woman. He found also that of this entire number, twenty-five per cent. of the cases were communicated innocently. Therefore, syphilis really is not a disease which necessarily implies dereliction of moral duty on the part of the individual. The general impression is that in order to have syphilis one must seek it, and seek it through the ordinary impure sources; but the syphilis among married women and nurses and the syphilis that is communicated in industrial pursuits, and what may be termed professional syphilis among surgeons, midwives, and dentists, must be placed in the category of *unmerited syphilis;* the number of cases of syphilis communicated in perfectly innocent ways is enormous.

In speaking of the duration of the contagious stage of syphilis, the reader of the paper referred more especially to mucous patches and syphilitic ulcerations, and, as he intimated, it is impossible to fix a definite limit which marks the end of the contagious activity of these lesions. Ordinarily, after two or three years we do not regard syphilis as contagious. Certainly cases of contagion have been reported as occurring from older lesions, but these are so very few that the danger of the contagion of syphilis after three years may be looked upon as infinitesimal. It may be said that the lesions of secondary syphilis, especially buccal lesions, are ultra contagious. We find, in tracing the origin of syphilis in married women, that an immense majority of the cases of syphilis have been conveyed not from the chancre, but from mucous patches. These patches have their origin and centre in the mouth. The blood of syphilis is also very actively contagious during the entire secondary period.

There is one possible mode of contagion which Dr. Bulkley omitted to mention,—viz., syphilis communicated by the saliva. Now, it is well known that the physiological secretions are not contagious; that is, saliva, the milk, the tears, and other physiological secretions do not, in a pure state, contain the virus, but they may serve as vehicles for the transmission of syphilitic poison; when the saliva becomes mixed with the secretions of the mucous patch in the mouth it is just as capable of conveying the disease as the secretions taken direct from the patch itself. It has been proved that syphilis has been conveyed from the patient to the operator by his placing an instrument between his teeth, which was moistened with the patient's saliva. A number of cases have been reported which have been due simply to the mixing of the secretions of the mucous patch with the salivary secretions in the process of tattooing. As I have said, the dentist is exposed to great possibilities of contagion. In common with most specialties, I almost always begin the treatment of syphilis by an examination of the patient's mouth and teeth, and ordinarily, if I do not find them in good condition, my first step is to send the patient to a dentist to have the teeth cleaned and the broken, jagged edges smoothed. I do this simply because I recognize, as others do, that in the treatment of syphilis it is necessary, in order to prevent the occurrence of mouth lesions, that the teeth and the gums should be kept in the best possible condition. I think it is the practice of most physicians who treat syphilis to pay special attention to the condition of the teeth, and it has sometimes occurred to me whether it was a proper thing for the physician in sending a syphilitic patient to a dentist to enlighten him as to the nature of the patient's disease; in other words, whether it be right or proper to expose the dentist to a possible source of contagion, which he might not recognize, without some intimation. We all know how close and intimate the confidence is between patients of this class and the physician, and I have concluded that, considering the infinitesimal danger to the dentist, it is not proper to violate professional confidence to that extent. But I certainly concur in the suggestion of the reader of this paper that dentists should make themselves familiar with the mucous membrane manifestations, or at least the buccal lesions of syphilis. These when once seen cannot fail to be readily recognized; that is, the typical mucous patch is so characteristic in its aspect and location, and in its course, that it may be recognized at a glance; and it is certainly for the protection of the dentist that he should be able to recognize such a possible source of danger when he is brought in contact with it. Fortunately the epidermis of the fingers and the hands is so thick that it constitutes a very effectual protection. I do not believe it is possible for the syphilitic poison to penetrate through the unbroken epidermis of the hand, and unless the dentist happens to have an abraded surface, hang-nails, or the nails cut too close, leaving an exposed point for the entrance of the poison, I do not think there is any possibility of his contracting syphilis by having the fingers come in contact with syphilitic virus.

One prophylactic measure which might be used with advantage in cases like those, and which probably would not interfere with the dentist's manipulations, would be to have the exposed fingers protected by rubber stalls; this would constitute perfect protection.

As regards the disinfection of instruments which are ordinarily employed by dentists, I would concur in the suggestion that strict asepsis of all instruments should be rigidly observed. My impression is that the ordinary smooth instruments, not rough instruments, can be thoroughly cleansed of all syphilitic virus by simply wiping them with a towel, or first moistening in alcohol and then wiping. The virus does not adhere so readily to a smooth surface as to a rough one, but it is better to err on the safe side and practise strict asepsis.

*Dr. E. A. Bogue.*—Perhaps I am in order in asking whether cold has its influence upon syphilitic virus; and, further, whether the use of oils of various kinds on the fingers would not be more or less of a protection, on the part of those who are operating, from such patients as Dr. Morrow has just spoken of?

*Dr. Meriam.*—There is one agent that we are in danger of losing sight of,—alcohol. It is, I know, used extensively for washing root-canals; but, I think, nearly all of us look on its mechanical value, when, in fact, it has a higher rank as a disinfectant.

Before antiseption received the attention that it does now, alcohol was used by surgeons to wash the parts after operations. The late Dr. Cabot, of the Massachusetts General Hospital, Boston, used to wash his fistula operations with it. We are informed that the ancient surgeons washed their operations with wine, and the good Samaritan thus seems to have had a practical knowledge of antiseptic treatment.

When we think of the specimens in our museum that it preserves we get an idea of its power. We should not have them did it not prevent the development of germs.

I am more and more inclined to the opinion that alcohol is all the value, beyond flavor, there is in many of the mouth-washes. Some physicians, I know, use it both as a spray and a gargle in diphtheria or diphtheritic sore throat.

It is the antiseptic *par excellence* for instruments, as it can be used without soiling, burning, or scenting the hands. Burs and files can be cleaned with it, and in operating below the gum I always dip the point of my instrument into a little that I have poured into one of the wells of my porcelain slabs, as I work, and often the points of excavators and burs. In excavating, the slight amount that adheres to the point of the instrument need not be wiped off. Those who prefer can use an essential oil or carbolic acid with it. Its non-poisonous, non-escharotic nature and the fact that it roughens the hands but slightly commend it to dental practitioners.

*Dr. Perry.*—Mr. President, you have called this a valuable paper. I would call it an invaluable paper. It happened to be my good fortune, many years ago, while living at Yonkers, to be a member of the Yonkers' Medical Association, by which means I was brought in contact with the physicians and surgeons who were members of that Association, and I particularly became well acquainted with Dr. J. Foster Jenkins, now dead, who was formerly secretary of the Sanitary Commission. For quite a number of years I was accustomed to observe the reports made by members of the medical fraternity of syphilitic cases, and while yet quite a youngster I became aroused to the dangers of the disease, and, in those early years I took the best means I could find at that time to guard against it. I have always felt that it is one of the greatest dangers we have to meet, and that there is a moral responsibility connected with it that the profession has never sufficiently considered. In those early years, before the bichloride of mercury became so generally used, it was my habit to have all instruments cleansed with a carbolic-acid solution, and in later years it has been the invariable custom in my office, when a patient is dismissed, to sweep aside all the instruments that have been used and take up another set, and the others are not again used until my lady attendant has cleansed and disinfected them and put them back in case. Several times I have been asked by visiting dentists, who happened to see that done, why I took all that trouble, and my answer has always been that, although I didn't feel really that there was so much danger of infection (because in all these years I have never seen a great many syphilitic cases), still I did not wish to take any chances; and so it has come to be my invariable rule that every instrument, even my pluggers, which are not supposed to touch the soft tissues at all, should be passed through a disinfecting fluid before being put in their places for use again. Dr. Jenkins impressed upon my mind the fact that syphilis could be conveyed through the blood of a patient. I was frightened and made to tremble by the presence of this danger; it has hung over me as a cloud; and yet such an accident has not occurred to my knowledge in my office. But there is a moral responsibility resting upon every one of us in regard to this matter, and I thank Dr. Bulkley for presenting the subject as he has to-night.

*Dr. William Frederick Holcombe.*—Mr. President, I was invited to be here to-night, and came because I wished to be present to hear the excellent paper of Dr. Bulkley, an authority that I consider one of the best in the world in his specialty, and I am happy to say so in his presence. About thirty years ago a gentleman came to me with a sore on the root of the first finger-nail. I looked at this sore and asked, "Have you hurt that in any way?" He said that he "didn't know that he had, but it had gradually come." After a very short time I saw him again; it developed into unmistakable syphilis. That gentleman went through a course of syphilis, was treated by several physicians, and escaped barely with his life. He said at the time that he was pricked by one of his instruments just below the nail. He was not aware that the instrument went into the flesh; but the probability is that there was a syphilitic sore in the patient's mouth and it inoculated the system with the syphilitic poison. In 1851 I was a pupil of Ricord, who has just died at the age of eighty-nine. He was an authority on syphilis for half a century; and among all the methods of contracting syphilis that he mentioned, I never heard him speak of this as one of the possible means for the contraction of this disease,—that is through the means of dental instruments. It shows that we are making progress at the present time. In these lectures that I heard in 1851, in the Garden of the Louvre Hospital, where he lectured, innumerable instances were given of the possible contraction of syphilis. They were so numerous that I wonder that we do not all contract it. I wonder that any of the doctors escape; and, as it is possible for a wound on the finger to become inoculated with the syphilitic poison in examining the mouth, the dentist is specially liable to contract it. I heard Ricord mention the fact that the application to the fingers of something of the character of lard or grease would probably often prevent the poison being taken into the system. I heard Dr. March, of Albany, advise his pupils, when making examinations of syphilitic patients, where they had hang-nails or any broken skin on the fingers, to always put some kind of ointment upon their fingers before operating. While going to the Medical Convention at St. Paul, I was talking with a gentleman from Columbus, Georgia, and he called my attention to a child who was on its knees in another seat, gnawing the back of the car-seat. He said, "I saw a man sitting down there a little while ago with some evidence of syphilis on the back of his neck;" and he said, "I believe that time and again persons contract syphilis by putting their heads back on the chairs where other persons having syphilis have reclined." I hardly ever sit down in a barber's chair without putting my pocket-handkerchief or something of that kind on the head-rest. We cannot, as a man said the other day, have a new chair every time, but we can have a new napkin for the back of the chair. The tumblers that the dentist and his patients drink out of may be the means of transmitting syphilis; if a man has a chancre on his lip he may give it to the person who drinks after him. I will state one case where two innocent persons, so far as I know, were the subjects of syphilis. This was about thirty years ago, in 1862. In a well-known family, an estimable girl, who was a companion of children, came to me with syphilitic symptoms and iritis. I asked to have the persons with whom she was living come to see me. They came. I asked to have another examination, and finally asked for a special consultation in the matter. I asked the girl in regard to her character, and she said that her character was perfectly pure. I asked her, finally, whether she had any sores on her lip. She said she had a bad sore about three months ago. There was a scar there. Her case was one of marked syphilis. She went through a course of treatment, the syphilis was cured, and she became a married woman, living in respectable society, but has had no children. She said at that time she had a man who wanted to marry her and that he had had a sore on his lip. The fact undoubtedly was that she got her syphilitic sore from him through kissing.

A lady came to me fully eighteen years ago who had terrible syphilitic iritis. She belonged to one of the best families. I found that her mother had a similar sore, also her aunt, and her grandmother. There was a young man courting one of the daughters, whom he had kissed and had inoculated the whole family.

*Dr. Kingsley.*—May I ask what is the moral of that,—that a man must not kiss his grandmother?

*Dr. Holcombe.*—He must not kiss anybody. I recollect an instance that occurred once on board some ship with Horace Greeley's wife. Her little child was taken up by a stranger and kissed. The mother took the child from him and said, "Sir, I do not allow you or anybody to kiss my child." She took her handkerchief and spat on it and rubbed the lips off. This kissing business is often perfectly terrible in results. Some nurses kiss a child on the lips, and kiss it on the nose and ears, and kiss it all over; and I have not the least doubt but that syphilis is conveyed to many innocent persons by this habit. I should like to ask Dr. Bulkley one question: if he believes that the dry powder of syphilitic chancre, having been rubbed off, like vaccine virus, and blown through a room, would, by being inhaled, be the means of communicating syphilis. I heard Ricord once say that many cooks would communicate syphilis to a whole family; the cook would have a sore on his lip and would not think anything about it, and he would taste the different dishes and put his fingers into them,—and all know the French cook has a habit of handling the meats with his fingers,—and many people undoubtedly have contracted syphilis from food handled by cooks. It was the old-fashioned way in hotels of having the towels on a roller for the common use of guests; and I remember once on a Scotch steamboat there was a tooth-brush for the whole crowd. I have no doubt that tooth-brushes sometimes used by several younger members of a family have been the means of communicating syphilis. I have had many patients come to me and say that they caught the disease from a water-closet; and I have no doubt that many cases of gonorrhœa and syphilis have been communicated by means of water-closets. I think they should be under direct sanitary inspection, just as much as the foul air that comes from the sewer. Not long ago I was in a cigar-makers establishment, and I noticed that every cigar-maker, when he was finishing the end of the cigar, wet the forefinger and thumb with his lips to point it nicely. I wondered at the time if he had a lip-chancre; and question if the disease has not been given to many persons in that way. I know of a case where, a few days ago, a lady kissed every female in the room, and one of the persons in that room I know had had syphilis. Some people make a business of kissing everything, kissing animals, birds, dogs, and cats, and it is very suggestive of the transmission of disease. The habit of putting money into the mouth, that some people have, is one that might lead to syphilitic infection. A butcher or grocer will often hold half a dozen bills in his mouth, and children and grown people, will put silver pieces and pennies in their mouths; and I have wondered that the habit has not been more productive of disease than we have observed. At all events, it is well to have our attention and that of the public called to the means of contracting syphilis and other diseases.

*Dr. Perry.*—Mr. President, may I ask one question? Dr. Bulkley alarmed me a little by his remarks in regard to the implantation of teeth. The dangers he spoke of existed, of course, before the time when antiseptics were used as they are now; but I have assumed that we could not now be in danger of conveying disease by implanting teeth. I have considered that there was no danger, at least to the patient, but I have felt that I could not really perform the operation without danger to myself; that I must run the risk that all surgeons run whose hands must be covered with the blood of their patients, a greater danger to me than to the patient. If in implanting teeth we have not rendered them as safe as we supposed by the disinfectants we have used, it is a serious matter. I have made about sixty implantations, and I have done it with a feeling of perfect assurance that there was no danger to the patient. Since Dr. Bulkley has made this statement about the risk attending the implantation of teeth, I would like to ask him if he considers there is any, provided the teeth are cared for as we care for them, in the matter of previous treatment and the attention to the details of thorough cleanliness?

*Dr. Dwinelle.*—Mr. President, I am exceedingly obliged, for one, to Dr Bulkley for the very interesting and suggestive paper he has read to us to-night. I have no doubt that it is based on the facts of the case, and I think they will find an echo in every one of our minds, and that the points made will be endorsed and will suggest many others similar. I think it was John Wesley who originated the expression that cleanliness was next to godliness. He put cleanliness first! I think the lesson given to us to-night should be appropriated by every one present. I have no doubt it has been, and that the hints given us will induce us to be more assiduous and careful in the respect indicated. It is incumbent upon us to be exceedingly cleanly in every department of our work. The matter of implanting teeth suggests the fact that John Hunter was among the first to transplant teeth, which he did successfully. In one instance he transplanted a tooth from the mouth of a servant girl into that of a lady of quality, who thereby became infected with syphilis, from which she lost her life. Subsequently the practice was discontinued until recently, when the similar process of implantation has taken its place. I have no doubt that the matter of the implantation of teeth has suggested to the mind of the operator in our day the unhappy results in John Hunter's case; but our apprehensions are relieved in this respect, from the fact that the teeth we use are always subjected to chemical disinfection, which renders transmission of any virus impossible. It has been my habit, when I have found a patient with syphilitic or other disease, to use a certain set of instruments for that patient exclusively. It were better still not to operate for such patients at all. If it were known to our patients generally that in a single instance we harbored a pronounced syphilitic patient, our practice would become exceedingly small. All our instruments should not only be kept clean, but they should be constantly disinfected. I remember one case, a very marked case, which I once treated. The patient had a perforation of the palatine arch, through which I could insert my little finger. I succeeded in closing the opening in the course of six months. The patient afterwards fell into the hands of an electrician, who made the hole larger than before. I finally closed it with a plate.

I once saw a well-authenticated case of transmission of syphilis in the fingers of a dental operator. We owe it to our patients, as well as to ourselves, to be ever watchful and on the alert in this matter, especially when new patients occupy our chair. Our experience has taught us that a little quiet quarantining in this direction can do no harm.

I am glad this subject has been brought up. As Dr. Perry has remarked, a good healthy fright on the subject will do us good.

*The President.*—Gentlemen, if none of you have anything further to offer on the subject, I will ask Dr. Bulkley to close the discussion.

*Dr. Bulkley.*—Mr. President, I will only answer some questions that have been asked. I think the comparative immunity of the physician and the dentist, the infrequency of the communication of the disease to themselves, is due to the frequent washing of the hands and the guarding of any sores that may be upon them.

I am asked by Dr. Bogue in regard to the application of grease to the fingers of the operator. I certainly think that is a most important protection in cases of exposure. Only last week I examined a case of chancre in the back of the throat, in the region of the tonsils, and in order to make the diagnosis clear I wished to feel it with the fingers. I rubbed my fingers with vaseline, and then felt for the chancre, and satisfied myself in regard to the nature of the sore, which proved to be a hard chancre, the primary lesion of syphilis. After wiping my hands with cotton and washing them I felt safe. Oils and fatty matter are good protectives against the virus. I could put a quantity of syphilitic virus on my hand and leave it there to dry over night, and I do not think there would be any danger as long as there was no broken surface; upon a healthy finger there is no danger. If an accident should occur by which the epidermis is punctured, I think a safe course would be to apply suction to the wound immediately. If a finger should be wounded, wash it instantly and suck it freely and thoroughly, spitting out the blood.

I am asked by Dr. Bogue about the efficacy of cold. I did mention cold, and said it would kill the virus. Undoubtedly cold, freezing, will kill the syphilitic germ.

The suggestion in regard to the use of alcohol is a very good one. I think I should have mentioned alcohol in connection with the bichloride of mercury and carbolic acid as destroyers of syphilitic poison. Pure alcohol is undoubtedly a thorough antiseptic.

*Dr. Perry.*—May I ask Dr. Bulkley how strong a solution of bichloride should be used?

*Dr. Bulkley.*—I hardly know. I have an idea that it is sometimes not used strong enough. I should think that less than one to a thousand, and certainly one to five thousand, would be useless.

*Dr. Perry.*—Would one to five hundred be unsafe?

*Dr. Bulkley.*—I hardly know. I would not suggest it. It is very difficult of determination, because we have no means of testing or verifying it. We are naturally unable to experiment on human beings, and animals will not take syphilis. You cannot inoculate a horse, dog, or cat. Monkeys have been thought to have been inoculated, but it is very doubtful. I am asked whether the dry powder of syphilis, when blown through a room, would convey the disease. I should say not. The quantity would be so infinitesimal that I think it would not convey the disease unless it came upon a wound. If taken into the lungs it would be thrown out. We know of no means of syphilitic inoculation except through an open wound.

In regard to the acquiring of syphilis innocently in water-closets, I only recently found one authentic case in literature. None of the old writers have given an authentic case. Cigars do give it. I have myself reported two cases where men had chancre of the lip from cigars. I saw a cigar-maker who ceased rolling cigars because his lips were so sore from mucous patches that he could not roll and moisten them any longer. They tell me that manufacturers are now very strict in cigar-factories, and dismiss a man when they find them rolling cigars on the lip; but formerly it was not so.

*Dr. Allan.*—Tobacco is a strong antiseptic.

*Dr. Bulkley.*—It does not prevent inoculation from a cigar.

In regard to planting and transplanting teeth, these cases that I speak of were all old, going back about one hundred years, to the time of John Hunter. I cannot find any recent cases. It is said that there have been new cases reported recently of syphilis conveyed by transplanting teeth, but I have not been able to find them. I suppose the modern method is to clean and render aseptic the teeth implanted.

*Dr. Perry.*—Yes.

*Dr. Bulkley.*—The periosteum is cleansed?

*Dr. Perry.*—Yes. I am very much exercised by the statement of Dr. Bulkley that a solution of bichloride one to five hundred might not be sufficient. The teeth I have implanted, and the teeth that Dr. Younger implanted have been subjected to a solution of bichloride from one to five hundred to one to one thousand; if that is not strong enough, we have been in greater danger than we dreamed of.

It has been assumed that in the use of bichloride, one to five hundred, we were perfectly safe in the performance of the operation.

*Dr. Bogue.*—Mr. President, it seems to me that there is a little bit of talking at cross purposes. Dr. Bulkley evidently does not understand that Dr. Perry is speaking of the implantation of teeth that may have been extracted from one to one hundred and fifty years back. The transplantation of teeth is a thing which is done immediately from patient to patient. Teeth that have been dried for years, I suppose, are beyond question safe; but transplanting from patient to patient within ten minutes is what I understand Dr. Bulkley to be speaking of.

*Dr. Bulkley.*—I presume, Mr. President, that the experience of dentists is that implanting teeth is like driving pegs of bone into bone,—they are absorbed,—that a tooth has no life when transplanted, but is utterly dead; then certainly it can be penetrated by your antiseptic; but whether a solution of one to five hundred is sufficient or not I cannot say.

*Dr. Perry.*—I never transplanted teeth freshly from one patient to another: never. I have no doubt that that can be done with safety in many cases, but I have never taken the risk. All the teeth I have planted have been first dried and then soaked in the bichloride.

*Dr. Morrow.*—I would say that there is hardly a possibility of the syphilitic virus retaining its potentiality for mischief after it has been dried for any considerable length of time. There is no danger whatever in the implantation of teeth as ordinarily practised. Certainly a tooth that had been dried for twelve months or longer could not possibly convey syphilis. So far as the transplantation of teeth is concerned, it seems to me that it would be a very reprehensible act on the part of the dentist to transplant a tooth from one individual to another unless he had satisfied himself that the former was not syphilitic. It is quite an easy matter to determine whether a patient is in the active stage of syphilis or not, and that should be determined before extracting the teeth and transplanting into another individual's mouth. That, it seems to me, is a very much readier means of solving the problem than any possible disinfection. I think bichloride one to five hundred is amply sufficient to destroy the germs of syphilis.

Dr. Howland moved a vote of thanks to Dr. Bulkley for his very valuable paper. Carried.

Adjourned.

S. E. Davenport, D.D.S., M.D.S.,
*Editor New York Odontological Society.*

